# Bjørn Ibsen: What Made Intensive Care So Critical?

**DOI:** 10.7759/cureus.67281

**Published:** 2024-08-20

**Authors:** Diego Ángeles-Sistac, Indalecio Morán-Chorro, Luis Morales-Quinteros

**Affiliations:** 1 Critical Care Medicine, Hospital de la Santa Creu i Sant Pau, Barcelona, ESP; 2 Critical Care Medicine, Vall d'Hebron Institut de Recerca, Hospital Vall d'Hebron, Barcelona, ESP

**Keywords:** critical care anesthesiology, jordi mancebo, bioethics, history of medicine, mechanical ventilation, critical and intensive care, bjørn aage ibsen, historical vignette

## Abstract

The COVID-19 pandemic underscored the critical importance of intensive care units (ICUs), a field institutionalized by Bjørn Ibsen during the 1952 polio epidemic in Copenhagen. Ibsen's groundbreaking innovations, including positive pressure ventilation and real-time physiological monitoring, laid the foundation for modern intensive care medicine. Trained in Denmark and the United States, Ibsen demonstrated the effectiveness of manual ventilation during the polio outbreak after successfully resuscitating a young patient, Vivi Ebert, which in turn led to the creation of the world's first multidisciplinary ICU at Blegdams Hospital.

This article explores the historical context and significance of Ibsen's contributions, tracing the evolution of the physiology of breathing from the early concepts of Vesalius and Hook to the widespread application of ventilation techniques. The establishment of the ICU introduced new ethical dilemmas, highlighting the delicate balance between prolonging life and maintaining patient dignity. Ibsen's legacy extends beyond medical advancements to the compassionate care he championed, a principle that remains a cornerstone of modern intensive care. This ethical complexity is a crucial aspect of the history of intensive care medicine.

## Introduction and background

The year 2020 will be remembered as the time when the COVID-19 pandemic not only thrust intensive care into the global spotlight but also significantly heightened public awareness about the life-saving support provided in these units. It was a moment of enlightenment when the world truly grasped the pivotal role of intensive care in healthcare, a concept that had been relatively unknown in medical history until then. 

This article is a tribute to the man who, amid a polio epidemic 71 years ago, played a transformative role in revolutionizing the management of the critically ill outside the operating theater. His invention of intensive care medicine stands as a testament to human ingenuity and the relentless pursuit of saving lives, inspiring us all.

The work of intensive care physicians is primarily defined by "saving lives" or "sustaining life" rather than diagnosing or curing particular diseases [1]. The core capability that underpins intensive care medicine is the ability to support temporarily and, in some cases, replace the function of multiple organ systems in the face of critical illness and injury [2].

Intensive medicine, one of the youngest medicine specialties, if not the youngest, was cemented upon the shoulders of Bjørn Ibsen, a brilliant and compassionate anesthetist who sought a way to replace the functions of the body when the physiological reserve, exhausted by disease or extensive damage, was unable to recover. 

**Figure 1:**
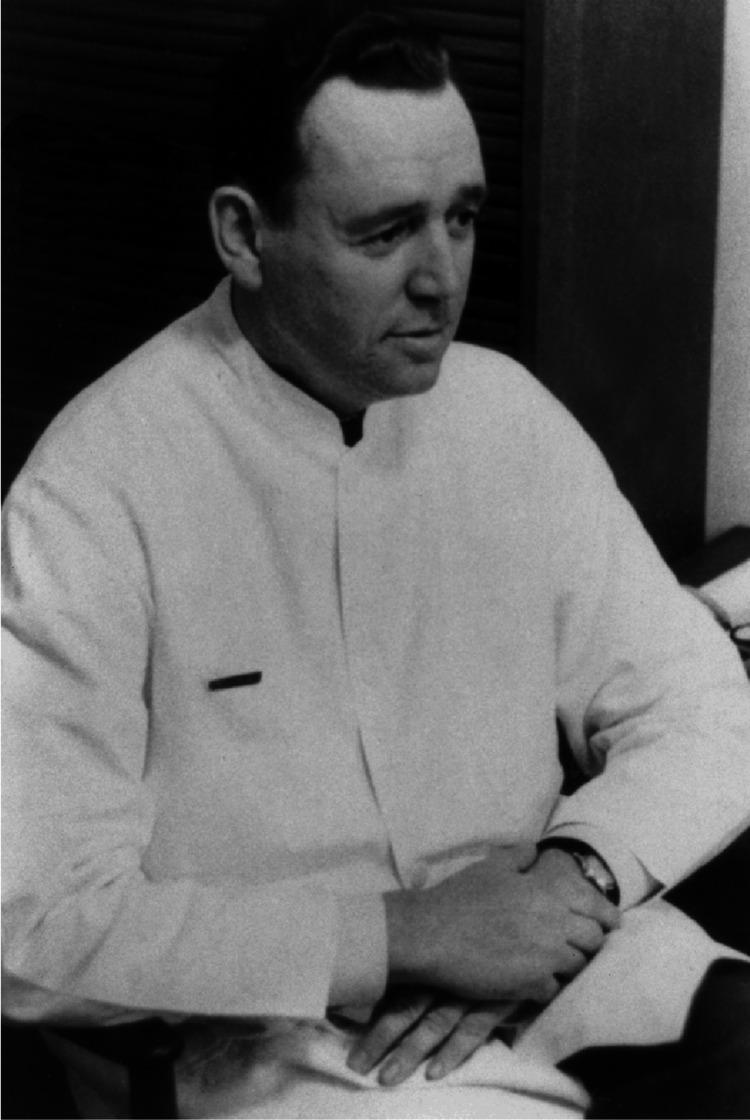
Dr. Bjørn Aage Ibsen Source: [6]. This image has been cited appropriately to its original source in accordance with fair use policies. Copyright/license: This figure has been adapted from Reference [6], which is an open-source article distributed under the terms and conditions of the CC BY 4.0 license. (https://creativecommons.org/licenses/by/4.0/)

The birth of intensive care units (ICU) is often dated back to August 1952, when Bjørn Ibsen (Figure 1) convinced his colleagues that tracheostomy, suctioning, and the around-the-clock application of positive pressure ventilation were necessary to save the lives of patients with paralyzing forms of poliomyelitis [1]. The new discipline blossomed in the wake of the 1952 Copenhagen polio outbreak [3-6].

History highlights Bjørn Ibsen's use of positive pressure ventilation (respiratory function replacement) as the cornerstone of intensive medicine. However, his contributions also encompassed three equally important landmarks: understanding physiology and measuring its effects in real-time, overcoming the logistical challenges of the most labor-intensive and technologically advanced specialty, and addressing a new bioethical paradigm where patients became entirely dependent on the tools of this new medicine.

## Review

A humble beginning

Bjørn Ibsen was born in Denmark on August 30, 1915, and obtained his medical degree at Copenhagen University in 1940. During those years, Denmark was less industrialized compared to other European nations. According to Ibsen's son Thomas, the Danish healthcare system was essentially comprised of three key figures: the doctor, the pharmacist, and the priest [4,5].

During his medical studies, Ibsen sampled multiple areas of knowledge: radiology, surgery, pathology, and gynecology. Ultimately, he traveled to the United States of America (US) in 1949 to become an assistant resident in the anesthesiology department at the Massachusetts General Hospital under H.K. Beecher [7]. Until then, in Denmark and most of the world, anesthesia was typically practiced by surgeons in the operating room [5]. 

While Ibsen was a creative problem solver in anesthesia and became one of the first six certified Danish anesthetists in 1950, there was no indication at the time that an extraordinary career awaited him [8]. 

**Figure 2:**
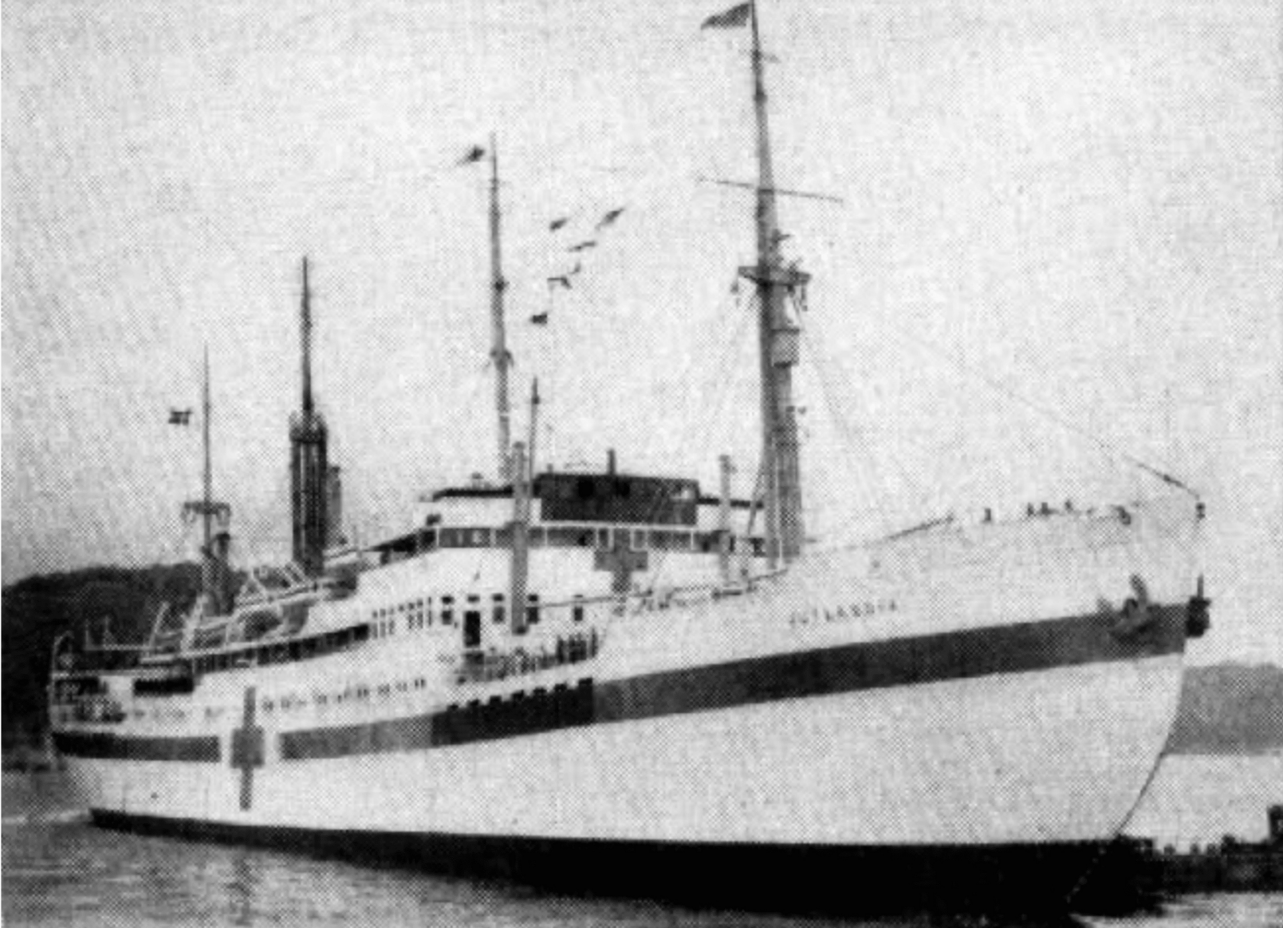
MS Jutlandia MS Jutlandia [9], the ship where Ingrid, Ibsen's wife, met Mogens Bjørneboe, changing Ibsen’s fate forever. This image has been cited appropriately to its original source in accordance with fair use policies. Copyright/license: This image is the work of a U.S. military or Department of Defense employee, taken or made as part of that person's official duties. As a work of the U.S. federal government, the image is in the public domain in the United States. MS: motor ship

A fateful trip taken by Ingrid, Ibsen's wife, in 1950 set the stage for Bjørn to become the legend he is today. During this trip, Ingrid met Mogens Bjørneboe, deputy to Hans Christian Lassen, head at Blegdams Hospital (Blegdams Fever Hospital). Their conversations piqued Bjørneboe's interest in Ibsen [5], leading to a series of events that would shape Ibsen's career (Figure 2).

In January 1952, at Blegdams Fever Hospital, a baby stricken with tetanus suffered from uncontrollable spasms. Mogens Bjørneboe recalled what Ingrid had told him about Bjørn and, as a last resort, asked for his help. Bjørn and Mogens decided to use curare to paralyze the baby and provide manual ventilation. Although the baby initially showed improvement, it unfortunately passed away [5]. Despite the unfortunate outcome, the viability of organ support-primarily positive pressure ventilation, in a non-surgical setting was demonstrated. However, efforts to restore breath to the breathless had been made long before Ibsen.

The breathing apparatus

The origins of mechanical ventilation can be traced back five centuries to Andreas Vesalius's seminal 1543 work, *De Humani Corporis Fabrica*. His studies include the first depiction of positive pressure ventilation: "But that life may be restored to the animal, an opening must be attempted in the trunk of the trachea, into which a tube of reed or cane should be put; you will then blow into this, so that the lung may rise again and take air" [10].

Nevertheless, it was a century afterward, in 1667, when Robert Hook showed the possibility of external ventilation to sustain life. In an experiment, he made cuts into a dog's chest wall and pleura and used bellows to generate a constant flow of gas through a dog's airway. He found that when said flow was arrested, the dog would agonize, but when the flow was restarted, the dog was resuscitated [3].

It was not until the late 19th century that medical ventilators were used in practice. At that time, these inventions were largely based on the accepted physiological principle that ventilation was a negative pressure process initiated by the diaphragm. Therefore, ventilators generate subatmospheric pressure around the patient's body to replace or augment the work done by the respiratory muscles. These bulky steel contraptions, which encased the patient's body, were dubbed the "iron lung" [5,10].

In 1876, Alfred Woillez built the first workable iron lung, which he called the "spirophore." Drinker and Shaw developed the first iron lung to be widely used in Boston in 1929, primarily to help polio victims breathe. Despite their spectacular nature, these machines were raucous, claustrophobic, cumbersome, expensive, and, most importantly, futile; about 80 percent of bulbar polio patients died comatose in the iron lung [4,10]. 

In 1949, at the Los Angeles County Hospital, physician Albert Bower and engineer Vivian Ray Bennett combined the mechanisms of the negative pressure tank ventilator with a positive pressure oxygen-supply system modeled for World War II pilots. The device decreased the mortality of severe polio from 79% to 17%. In 1950, their results were published in a lesser-known medical journal and went largely unnoticed (the results were also harshly negated, implying that the patients Bower and Bennet had seen were not as ill as they had reported). However, Ibsen, who had returned to Denmark in February 1950 after completing his one-year fellowship in Boston, read it and immediately understood its significance [5-6].

Bjørn and Vivi’s day

In the autumn of 1952, seven years after liberation from Nazi occupation, Copenhagen faced one of the most severe polio epidemics in history. Over 2,700 patients were admitted between July and December, plunging the city into a major health crisis. Of these, 866 patients experienced paralysis, including 316 who suffered from respiratory or pharyngeal paralysis [4,5,8].

At the height of the epidemic, 50 patients a day were being admitted to the Blegdams Hospital, many with respiratory muscle or bulbar paralysis. Too weak to cough, many patients drowned in their own secretions. Mortality in these patients was exceedingly high, reaching 80% [4,5,8,10].

The number of patients with respiratory failure was higher than in any other European country. The Blegdam Hospital, tasked with treating poliomyelitis, had only one tank respirator or iron lung and six cuirass respirators available to accommodate the daily admission of six to 12 patients presenting with respiratory failure [2-5,8].

Considering the bleak outcome of the epidemic, Blegdam Hospital's Chief Physician, Dr. Lassen, sought new ideas to fight the polio onslaught. Upon the suggestion of his deputy Bjørneboe, he contacted Bjørn Ibsen [4,5,8].

Never having treated polio on such a scale before, Ibsen recommended a new approach to ventilation, one that he had studied in the US and had performed previously on other types of patients: "bagging." That is, he intended to treat respiratory failure with a tracheostomy and positive pressure ventilation with the help of an air-filled bag [4,5,10].

Ibsen's bagging was not widely accepted at the time as it contradicted established human physiology. However, Ibsen argued that polio was being treated incorrectly with iron lungs, which resulted in inefficient ventilation. Patients with bulbar polio often faced hypercarbia, hypertension, and diaphoresis in their final moments, symptoms attributed to renal failure and thought to be the result of polio. Contrary to this belief, Ibsen, drawing on his experience in Beecher's lab in Boston, suggested these symptoms were due to carbon dioxide retention. He proposed that optimizing ventilation with positive pressure and using a carbon dioxide absorber could improve outcomes. Although Lassen initially rejected this approach, Ibsen soon had the opportunity to demonstrate the efficacy of his method [3-5,10,11].

On August 26, 1952 (Bjørn Ibsen Day), Vivi Ebert, a 12-year-old girl, presented with four-limb paralysis, respiratory failure, and bulbar palsy associated with acute poliomyelitis. After convincing Lassen, Ibsen was allowed to try his new ventilating technique the next day. A rubber-cuffed tracheostomy tube was inserted into Vivi, and Ibsen tried to ventilate her manually. Nonetheless, she was extremely agitated and fought the ventilation; her vital signs were worsening, and onlookers of the Ibsen method began to disperse, having decided the fatal outcome beforehand. Ibsen then administered thiopentone (a sedative) to calm the patient and even transfused blood to treat hypotension; all of this allowed for easier ventilation. After this, Vivi began to resuscitate. Being considerably improved by this treatment, she was transferred to a body-cuirass respirator to provide negative pressure ventilation for her continuing breathing inadequacy. Still, with the cuirass, she soon relapsed (again, carbon dioxide retention). Ibsen had to intervene over and over again with bag ventilation, reversing her deterioration [2,4,5,12,13].

Ibsen later acknowledged that if his attempt to resuscitate Vivi Ebert in August 1952 had failed, as it nearly did, he would not have been afforded a second chance. Bjørn Ibsen's demonstration established a new treatment for the respiratory complications of polio, which was promptly embraced at the Blegdam [5,10,11]. Since then, sedation and ventilation have remained inseparable twins in the treatment of acute respiratory failure.

Ibsen had successfully kept alive one polio patient with respiratory failure, and the Blegdam hospital was overrun with patients like Vivi. How would he manage such a labor-intensive procedure? And how would he be able to tell his methods were working?

The first intensive care unit

Delivering care to all the polio patients at the Blegdam Fever Hospital was a major logistical problem. There were no positive pressure ventilators, so the patients had to be "handbagged" individually. At the height of the epidemic, tens of patients with signs of impending respiratory failure were admitted onto the wards each day. During the summer of 1952, the staff treated more children with severe polio than they had in the previous decade [4,8,10].

**Figure 3:**
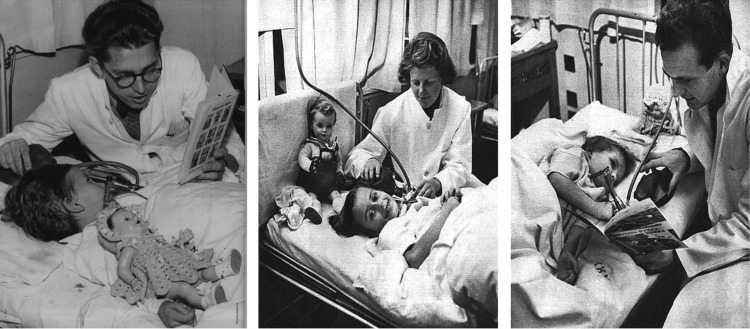
Student ventilators Ventilation of patients by medical students during the polio epidemic [6] This image has been cited appropriately to its original source in accordance with fair use policies. Copyright/license: This figure has been adapted from Reference [6], which is an open-source article distributed under the terms and conditions of the CC BY 4.0 license. (https://creativecommons.org/licenses/by/4.0/)

The hospital recruited physicians, dentists, and medical students to surmount this labor-intensive challenge. Medical students were drafted in and paid US $6 (today) per hour. 250 medical students came in daily and worked shifts with 35-40 doctors [2,5]. By the end of the epidemic, approximately 1,500 students had provided manual ventilation for a total of 165,000 hours. The effort consumed 250 ten-liter breathing gas cylinders each day at one time, and up to 70 patients required simultaneous, around-the-clock ventilation (Figure 3). The students bagged in shifts, pausing for meals and cigarettes; they socialized with their patients, played games together, and even learned to read their lips; sadly enough, they also mourned when treatments failed [4,8,10]. Amazingly, not a single volunteer or other person directly involved in the care of patients at the Blegdam would contract polio [5].

To adjust ventilation, the first account of constant invasive metabolic and vital sign evaluation was conducted; thanks to the invention of the first pH, partial pressure of carbon dioxide (PCO2), and partial pressure of oxygen (pO2) electrodes by Astrup, Siggard-Anderson, and Severinghaus, blood gas analysis was possible (before that, primitive sensors, some repurposed from the US Army Air Force and anesthesia applications, were used to signal falling blood oxygen and rising carbon dioxide) [2,5,14].

To maintain a standard of care and maximize treatment efficiency for multiple patients with acute respiratory failure, Ibsen urged the hospital to enforce three measures: centralize all these patients into one location, create dedicated nursing teams, and review patients with a multidisciplinary team. This led to the conceptualization of the ICU as we know it today [5,8,10].

After incorporating Ibsen's ideas into the Blegdam Hospital, mortality dropped dramatically from 87% to approximately 40% almost overnight. The initial three months of this new treatment system were estimated to have saved 100 lives [8,10,11].

The team at Blegdam Hospital implemented a multifaceted approach to patient care, encompassing preventive, rehabilitative, and reevaluation strategies. They instituted a comprehensive triage system to identify impending respiratory failure early. Dr. Ibsen and his colleagues went beyond hospital walls, assessing potential polio cases in surrounding communities and providing ventilation during transport to Copenhagen when necessary. Inside the hospital, attention was given to nutritional support and prevention of bedsores. Patients benefited from educational resources such as teachers, books, and music, promoting mental and emotional well-being. In cases where patients succumbed to their illnesses, autopsies were routinely performed to determine causes of death and refine treatment protocols [5,8,10,11].

After the epidemic, Ibsen and his staff monitored and treated all types of medical and surgical cases with respiratory or circulatory problems 24 hours a day [3,8]. In an unexpected turn, Henning Sund Kristensen took over the running of this new model ward (Lassen had not supported Ibsen taking this job) [2,15].

Following his tenure at Blegdam, Bjørn Ibsen moved to the Municipal Hospital on August 1, 1953, where he inaugurated the world's first multidisciplinary ICU. This pioneering model of care quickly gained global acceptance. Modern "intensive care units" or "shock wards" were subsequently established in the US, first at the Los Angeles County General Hospital and later at the Baltimore City Hospital. By the late 1950s, ICUs were present in 25% of American hospitals, encompassing over 300 beds nationwide [4,15,16].

The success of the ICU had inadvertently brought an enormous and conflicting dilemma. Organ function replacement was no longer theoretically feasible; it was now a standard of care. Nevertheless, how much should physicians replace, and for how long?

Vivi Ebert exemplified these challenges. She required continuous mechanical ventilation until January 1953 and left Blegdam in 1959, enduring a seven-year recovery. She was quadriplegic and relied on others for most daily needs like eating and toileting; she slept on a ventilator under medical supervision. Tragically, Vivi passed away at 31 in 1971 from sepsis secondary to pneumonia [4]. Vivi had been the first ICU patient and, for all accounts, the first post-ICU victim, and, like her, many patients did not regain respiratory function, required long-term ventilation, and were heavily dependent for the rest of their lives [2,5].

To ventilate or not to ventilate: that is the question

At Blegdam, the lights were turned off at night to give the patients some rest while the volunteers quietly ventilated them manually. In the morning, the students who served as human ventilators faced the horrifying realization that some of the patients they had been helping to breathe had died through the night [5]. No matter how innocent the nature of the "mistake," it revealed a broader and more sobering truth: some patients were beyond the reach of effective ventilation, and some perhaps should never have been ventilated at all.

On August 24, 1974, Ibsen declared in an interview with Stenthoft, a radio journalist, that in some cases where ICU treatment had been proven ineffective or could be considered harmful, it would be "more humane to give morphine, peace, and comfort." Moreover, Ibsen confessed to removing patients from the ventilator when their illness was, in his opinion, insurmountable [4]. 

After the interview, Christian and conservative groups vehemently criticized Ibsen, accusing him of "actively euthanizing patients" and even of "murder," and there were expectations of criminal charges. Nevertheless, Ibsen was never formally charged with any crime [4].

Ibsen's words on helping patients die when intensive care became futile were justifiable and backed by hard evidence, particularly after witnessing the lasting effects of the ICU.

After successfully treating the acute presentation of polio, a subset of patients remained unable to breathe independently. By October 1953, more than a year after Vivi Ebert's treatment, 20 out of the original 318 patients cared for by Ibsen's team still required continuous ventilation at Blegdam Hospital. These patients became known as "chronic respiratory patients," individuals whom medicine continues to struggle to understand and adequately treat [4, 17-19].

As additional intensive therapies are applied in the ICU, patients become increasingly vulnerable. Prolonged dependence on organ support, sedatives, and paralysis can lead to a condition known as "chronic critical illness." Patients may develop muscle weakness, experience delirium, become more susceptible to infections, and have a shorter life expectancy [17-19]. Ethical management of their care becomes crucial at this stage. However, many patients are unconscious and legally deemed incompetent to make decisions. Given the immediate consequences of decisions made by intensive care doctors, determining what is best for the patient may not be as straightforward as it should be [2,20,21]. Notwithstanding, Ibsen consistently prioritized maintaining patients' dignity, even in complex ethical situations.

Today, most patients who survive and leave the ICU will endure this chronic critical illness, or "post-ICU syndrome." Many of them will have some degree of neurological deficit, will experience post-traumatic stress disorder, anxiety, or depression, or will become so dependent on others that they might never be able to work again. Up to 50% of patients with post-ICU syndrome will succumb within a year [17-19]. 

The controversy surrounding the 1974 Ibsen-Stenthoft interview gave way to a modern reevaluation of bioethics, including an address from the Pope on life support ethics, scientific acceptance of brain death, and landmark legal decisions regarding dependence on vital function replacement [4]. "...at the beginning of intensive therapy, it was a problem to keep the patient alive; today, it has become a problem to let him die." - Bjørn Ibsen, 1975 [4].

The last of Bjørn Ibsen’s days

Few innovations in the history of medicine have been so immediate and decisive as Bjørn's ventilation method. In the absence of a polio vaccine, human-mediated positive pressure ventilation, with all its quirks and faults, reduced bulbar polio mortality from 87% to 11% within a year [4,5]. 

Ibsen was clinically successful but struggled to gain professional recognition. This is associated with two factors: first, most descriptions of his personality do not portray him as ambitious or fame-seeking, and second, an ongoing feud with Lassen impeded his career progress. As a result, Ibsen was not appointed chief of the first ICU. Furthermore, Lassen later published the achievements from the Blegdam experience in The Lancet under his name without acknowledging Ibsen [13,14]

After Blegdam, Ibsen ventured into the study of hemodynamics and pain treatment; he even took more pride in his discoveries about shock than in his groundbreaking work in intensive care [7,8,16].

In his later years, certainly enough with the ICU still lingering in his mind, Ibsen told his children, "I am not afraid of dying; I am only afraid of how" [4]. Fortunately, fate granted him a peaceful end. On August 7, 2007, while taking a morning stroll in his garden, Bjørn Ibsen passed away [8].

Dr. Bjørn Ibsen was a respected physician, firmly believing in the merits of clinical observations and preserving patient dignity in health and illness. His students were surprised by the egalitarian relationship he maintained with them. Admired by his peers for his astuteness and ingenuity, he was an even-tempered man, discreet and unassuming by choice. He humbly attributed his successes to chance and coincidences [4,5,8]. Curiously enough, Bjørn Ibsen's scientific achievements, rooted in observation and methodical study, were marked by creativity, improvisation, and adaptability, much like the rhythms of his favorite musical genre: jazz [5].

## Conclusions

Survival for intensive care patients worldwide has been improving year on year. This improvement is despite a trend to admit patients with more comorbidities to intensive care. However, exploring quality of life and long-term outcomes should be prioritized for the future of critical care medicine. Our responsibility is to make the ICU a compassionate and respectful place, particularly at the end of life, adding new missions and values to our field.

Bjørn Ibsen's most significant contribution to medicine was undoubtedly the creation of the ICU. However, if there is one lesson to be learned from the father of intensive therapy that goes beyond the brilliance of his inventions or discoveries, it is the importance of preserving a patient's dignity, regardless of the disease.

The first account of positive pressure ventilation might be traced back to God delivering the "breath of life" to instill the human body with a soul-filled vitality. Nevertheless, Bjørn Ibsen taught other physicians how to administer it themselves.

This article pays tribute to the brave intensivists who were tirelessly battling the COVID-19 pandemic on the frontlines. Specifically, it wishes to honor the late Dr. Jordi Mancebo, former Director of the Intensive Care Unit at the Hospital de Santa Creu i Sant Pau in Barcelona, Spain, who dedicated his professional career primarily to the study of respiratory failure and the use of mechanical ventilation. His mastery in medicine consistently exemplified the principle of putting the patient's interests first, following in the footsteps of Bjørn Ibsen.
